# Field Application of Tea Volatiles Mediating the Selectivity of *Aleurocanthus spiniferus* on Four Tea Cultivars

**DOI:** 10.3390/plants14172653

**Published:** 2025-08-26

**Authors:** Zhifei Jia, Xiaoyu Ge, Yanan Bian, Kai Song, Dandan Li, Dapeng Song, Shibo Ding, Yongyu Xu, Zhenzhen Chen

**Affiliations:** 1 State Key Laboratory of Wheat Improvement, College of Plant Protection, Shandong Agricultural University, 61 Daizong Street, Tai’an 271000, China; jiazf0525@163.com (Z.J.); gexiaoyugongzuo@163.com (X.G.); byn9810@163.com (Y.B.); 17853527051@163.com (K.S.); lidan@sdau.edu.cn (D.L.); xuyy@sdau.edu.cn (Y.X.); 2 Tea Research Institute, Rizhao Academy of Agricultural Sciences, No. 142 Haiqu West Road, Donggang District, Rizhao 276500, China; sdp20073882@163.com (D.S.); rzcksd@163.com (S.D.)

**Keywords:** *Aleurocanthus spiniferus*, synergistic bait trap, volatile components, plant-based attractants, host selection, field trials

## Abstract

Orange spiny whitefly (*Aleurocanthus spiniferus* Quaintance) is a major pest with economic significance to tea plants, as both nymphs and adults suck plant sap and contribute to the development of tea sooty mold. The occurrence of this pest varies considerably among different tea cultivars, even within the same plantation. This study aims to characterize the bioactive constituents of tea volatiles mediating *A. spiniferus* host selection, and leverage these semiochemicals to develop effective field trapping systems. Through field investigations and Y-tube olfactometer tests, we identified two highly preferred tea cultivars (‘Huangjinya’ and ‘Fuding white tea’) and two cultivars (‘Baiye No. 1’ and ‘Longjing 43’) that were not preferred. Behavioral assays and gas chromatography-mass spectrometry (GC-MS) analysis revealed four attractive compounds [hexanol, (*E*)-2-hexenal, linalool, and (*E*,*E*)-*α*-farnesene] and two repellent compounds [nonanal and (*Z*)-3-hexenol] in the volatile emissions of the four cultivars. A hexane solution (10 µL) of nonanol, (*Z*)-3-hexenol, linalool, and (*E*,*E*)-*α*-farnesene at a concentration of 100 µg/µL was able to elicit an obvious electrophysiological (EAG) response. In field trials, the synergistic bait trap equipped with two types of attractants, 500 µL of hexane solution of the mixture of linalool and (*E*,*E*)-*α*-farnesene (3:1, *v*/*v*), and the mixture of linalool and (*Z*)-3-hexenol (3:1, *v*/*v*) at the concentration of 100 μg/μL, showed significantly higher attractant efficacy and selectivity. Overall, this study indicates that tea volatiles play a crucial role in the host selection of *A. spiniferus*, and the synthetic mixtures of tea volatiles have the potential to be developed as commercial plant-based attractants for adult *A. spiniferus*. This study contributes to the development of sustainable, environmentally friendly management strategies for a pest that is challenging to prevent and control.

## 1. Introduction

Tea, *Camellia sinensis* (L.) Kuntze (Theaceae) is widely grown in tropical and subtropical regions, including China, Kenya, and Japan [1]. Tea not only has a unique aroma and flavor, it also has several medicinal values. Its phytochemicals can prevent and treat metabolic disorders (e.g., diabetes and obesity), digestive dysfunction, malignancy, and cardiovascular diseases [2]. The roots, stems, leaves, and flowers of tea trees contain diverse bioactive compounds. Key phytochemicals include flavonoids (tea polyphenols, catechins), methylxanthines (caffeine), amino acids (theanine), phenolic esters, tannins (tannic acid), pigments (theaflavins, thearubigins), and polysaccharides, all of which can be extracted for industrial applications [2,3]. The tea saponins extracted from tea leaves and tea seeds have the effects of antibacterial, hemolysis, anti-inflammatory, inhibition of alcohol absorption, anti-oxidation, and soil remediation abilities [4,5,6,7,8]. The average price of crude tea seed oil is the lowest of the vegetable oil feedstocks, but its quality is similar to that of vegetable oil [9].

Orange spiny whitefly, *Aleurocanthus spiniferus* (Quaintance) (Hemiptera: Aleyrodidae), is a major pest of tea plants [10]. Adults typically gather to feed on tea shoots and lay their eggs there. While the first instar nymphs remain active, all other nymphal stages are stationary and feed on the tea leaves, directly affecting the yield and quality of tea plants. Additionally, the excrement from this pest provides nutrients for the development of tea sooty mold (*Neocapnodium theae* Hara), which reduces photosynthesis in tea leaves and inhibits plant growth [11]. *Aleurocanthus spiniferus* is widespread in East and Southeast Asia, such as China, Indonesia, and Japan [12,13,14]. Currently, *A. spiniferus* is often controlled with chemical pesticides [15], but only 7 pesticides are approved for European Union (EU) tea cultivation [16]. This scarcity increases the risks of banned pesticides appearing in tea, as confirmed by EU Rapid Alert System for Food and Feed (RASFF) alerts. Recent notifications detected three prohibited chemicals in black tea: fenobucarb, dinotefuran, and tolfenpyrad [17,18,19]. Critically, chlorpyrifos, thiacloprid, and imidacloprid are banned in the EU due to proven harm to humans, animals, and ecosystems. These hazardous pesticides remain used in non-EU tea farms, posing health threats via imported tea [20], highlighting the urgent need for safer and more effective ways to prevent and control them.

Herbivorous insects need to locate and select host plants in order to find mates, resources, and oviposition sites, and olfaction plays a significant role in this process [21,22]. Adults can specifically detect volatile organic compounds (VOCs) emitted by plants, allowing them to discriminate between different types of resistant plants or cultivars on the basis of the types and proportions of VOCs present [23,24]. Generally, repellent components that discourage herbivorous insect infestation are present in VOCs from non-host plants or resistant cultivars [25]. For example, *Rosmarinus officinalis*, a non-host plant of *Ectropis obliqua* (Lepidoptera: Geometridae), contains repellent compounds [26]. The active ingredient limonene in extracts from celery, a non-host plant of *Bemisia tabaci* (Hemiptera: Aleyrodidae), had an inhibitory effect on egg laying [27]. Linalool exhibits both direct and indirect defensive properties, such as insecticidal and repellent effects (e.g., *Aedes albopictus* and *Aedes aegypti*) as well as the ability to inhibit egg laying (e.g., *Manduca sexta*) [28]. (*E*,*E*)-*a*-farnesene offers indirect defense against various insect pests [29]. Conversely, one of the main reasons that insects locate host plants or susceptible cultivars is due to mixtures of attractive plant volatile components [30,31,32]. For example, the volatile compound, (*Z*)-3-hexenyl-acetate, mediates *Spodoptera frugiperda* (Lepidoptera: Noctuidae) host and oviposition preferences on maize [33]. Pests of the Tephritidae family, such as the melon fly (*Zeugodacus cucurbitae*), locate host plants by antennal perception of common volatiles in cucurbit plants, including decanal and benzaldehyde [34].

The influence of volatile organic compounds (VOCs) on insect host selection behavior has enabled the successful application of specific compounds for population monitoring and integrated pest management (IPM) [27,35,36]. A binary repellent that contains 1,8-cineole and dimethyl disulfide (DMDS) has been applied using a slow-release method to combat *Empoasca onukii* (Hemiptera: Cicadellidae) in the field [37]. Attractants for *E. onukii*, such as (*Z*)-3-hexenol, (*E*)-2-hexenal, (*E*)-ocimene, geraniol, linalool, and (*Z*)-hexenyl acetate, have also been used in tea plantations [35]. The application of (*Z*)-3-hexenol enhanced direct and indirect plant defenses against tea geometrid *E. obliqua* [38]. Additionally, (*E*)-2-hexenal can lure *E. onukii* into pheromone-baited traps [39]. (*Z*)-3-hexenol, (*E*)-2-hexenal, and linalool have been developed as attractants for *Empoasca vitis* (Hemiptera: Cicadellidae) [39]. Hexanol serves as an effective attractant for the Haematophagous insect, *Nyssomyia neivai*; additionally, it can indicate a blood meal source for females [40,41]. Nonanal is a significant attractant for Hemiptera insects such as *Orius similis* (Hemiptera: Anthocoridae) [42]. In summary, VOCs play an important role in IPM, and further exploration of their utilization is warranted.

‘Huangjinya’ and ‘Fuding white tea’ are more susceptible to *E. onukii* and *Toxoptera aurantia* [43,44,45]. In contrast, ‘Baiye No. 1’ and ‘Longjing 43’ are commonly known resistant cultivars [46]. If VOCs that potentially mediate host selection are identified in these four tea cultivars, they could serve as effective plant-derived attractants for pest management and monitoring. To explore the resistance characteristics of these four tea cultivars, this study evaluated the population dynamics of *A. spiniferus* and assessed their selection preferences for each cultivar. Differences in volatile components among the four cultivars were analyzed using gas chromatography-mass spectrometry (GC-MS). The odor components in tea volatiles, which may play a crucial role in the host selection behavior of adult *A. spiniferus*, were examined through EAG and behavioral assays. Lastly, these compounds and their mixtures were tested as attractants in various tea plantations over two years to identify the most effective options.

## 2. Results

### 2.1. Population Dynamics of A. spiniferus on Four Tea Cultivars

**Figure 1:**
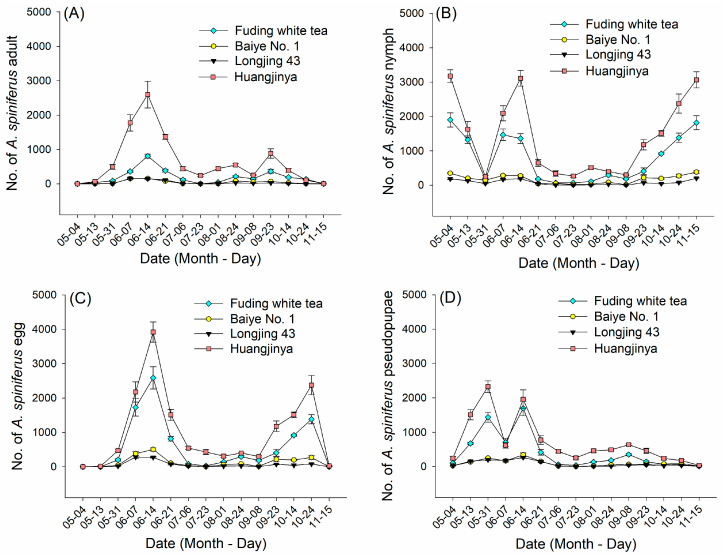
Population dynamics of *A. spiniferus* on four tea cultivars (‘Huangjinya’, ‘Fuding white tea’, ‘Baiye No. 1’, and ‘Longjing 43’) in Tai’an in 2022. (**A**) adults; (**B**) nymphs; (**C**) eggs; (**D**) pseudopupae. Abundances are shown as mean ± SE (*n* = 4). Bars indicate standard error.

The peak flight season occurred in June 2022. The number of *A. spiniferus* adults and eggs peaked on 14 June 2022, with significant variation in infestation levels among different cultivars (adults: *F* = 34.71, *p* < 0.001; eggs: *F* = 63.34, *p* < 0.001). The number of adults on ‘Huangjinya’ was significantly higher than that of ‘Fuding white tea’, ‘Baiye No. 1’, and ‘Longjing 43’ (Figure 1A). Egg abundance showed the same trend (Figure 1C). On 14 June, nymph abundance showed significant differences among different cultivars (*F* = 102.16, *p* < 0.001) (Figure 1B). Pseudopupae abundance on ‘Huangjinya’ and ‘Fuding white tea’ peaked on 31 May and 14 June, while the number of pseudopupae on ‘Baiye No. 1’ and ‘Longjing 43’ was relatively low (Figure 1D). Based on nearly a year of investigation, it was tentatively determined that the selectivity of *A. spiniferus* among the four tea cultivars in the same plantation environment was as follows: ‘Huangjinya’ > ‘Fuding white tea’ > ‘Baiye No. 1’ > ‘Longjing 43’.

### 2.2. Preference of A. spiniferus Among Four Tea Cultivars

**Figure 2:**
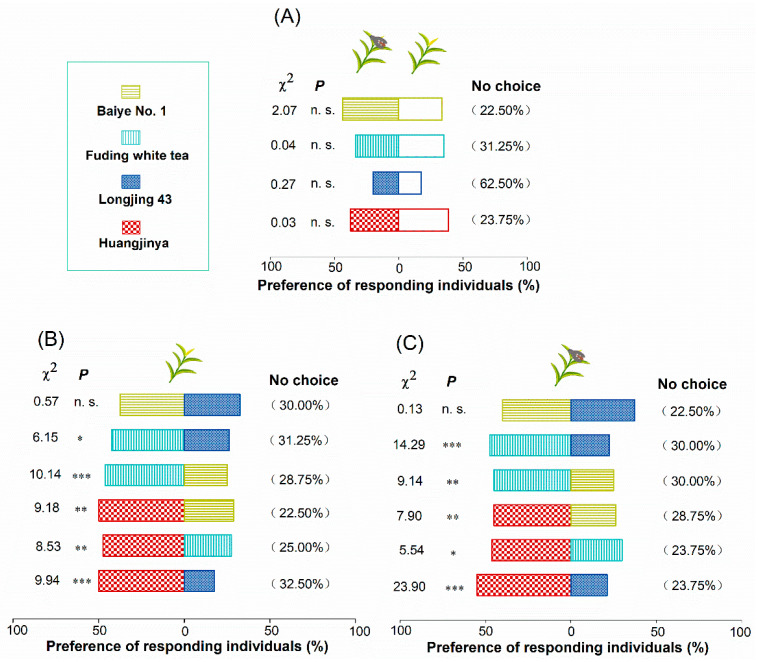
Responses of *A. spiniferus* adults to four tea cultivars in the Y-tube olfactometer. (**A**) Preference of *A. spiniferus* adults for healthy and whitefly-infested tea shoots. (**B**) Responses of *A. spiniferus* adults to four healthy tea cultivars in the Y-tube olfactometer. (**C**) Responses of *A. spiniferus* adults to four whitefly-infested tea cultivars in the Y-tube olfactometer. Numbers in brackets represent the percentage of *A. spiniferus* that were not selected. Asterisks and n.s. indicate significant (* *p* < 0.05, ** *p* < 0.01, *** *p* < 0.001) and non-significant preference between the two tea cultivars by chi-square test, respectively.

The results showed that the pre-infection of *A. spiniferus* nymphs statistically did not significantly influence the preference of the subsequent adult *A. spiniferus* (Figure 2A). The adult *A. spiniferus* showed significantly higher preference for the ‘Huangjinya’ cultivar compared to the other three. When ‘Fuding white tea’ was compared with ‘Baiye No. 1’ and ‘Longjing 43’, the adults tended to choose ‘Fuding white tea’. There was no significant difference in the preference of adult *A. spiniferus* between ‘Baiye No. 1’ and ‘Longjing 43’ (Figure 2B,C).

### 2.3. Volatile Profiles of Tested Tea Cultivars

A total of 13 compounds were identified, with 9 being common across all four cultivars. The content of hexanol (*F* = 10.86, *p* = 0.003), (*E*)-2-hexenal (*F* = 4.49, *p* = 0.04), and 7-methyl-heptadecane (*F* = 7.16, *p* = 0.012) exhibited significant differences among the volatiles from the four cultivars. Levels of hexanol and (*E*)-2-hexenal were higher in the resistant cultivars ‘Baiye No. 1’ and ‘Longjing 43’ compared to susceptible cultivars. Due to the inconsistency between the changes in 7-methyl-heptadacane content of four tea cultivars and the selective biological phenotype of *A. spiniferus*, this compound was excluded from this study. Additionally, linalool was only found in the susceptible ‘Fuding white tea’. Nonanal was absent in the resistant ‘Longjing 43’. The compound (*E*,*E*)-*α*-farnesene was only contained in the susceptible ‘Huangjinya’, while (*Z*)-3-hexenol was absent in the ‘Huangjinya’ (Table 1). In summary, we selected six compounds [hexanol, (*E*)-2-hexenal, (*Z*)-3-hexenol, linalool, nonanal, and (*E*,*E*)-*α*-farnesene] that varied between the four tea species for further testing.

### 2.4. Electroantennogram (EAG) Responses Elicited by Synthetic Compounds of Tea Volatiles

**Figure 3:**
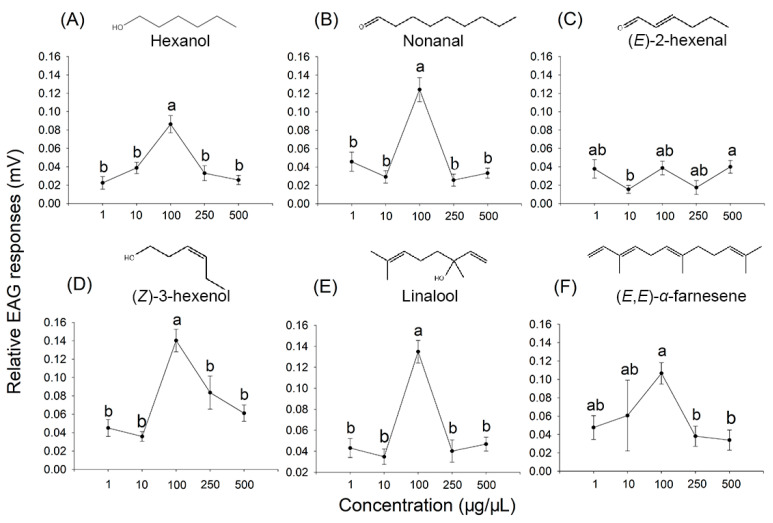
EAG responses of adult *A. spiniferus* to 10 μL of hexane solution of hexanol (**A**), nonanal (**B**), (*E*)-2-hexenal (**C**), (*Z*)-3-hexenol (**D**), linalool (**E**), and (*E*,*E*)-*α*-farnesene (**F**) at different concentrations (1, 10, 100, 250, and 500 μg/μL). Relative EAG responses are shown as mean ± SE (*n* = 18). Bars indicate standard error. Different letters above bars indicate significant differences in relative EAG responses (mV) at different concentrations, *p* < 0.05, one-way analysis of variance.

**Figure 4:**
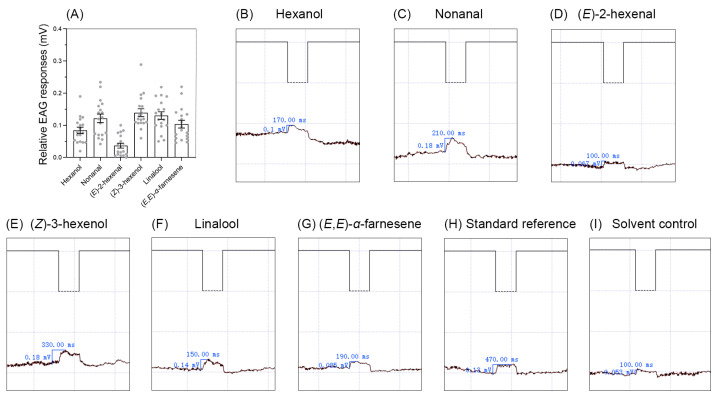
EAG responses (**A**) and examples of EAG recordings obtained with 10 μL of hexane solution of compounds at the concentrations of 100 μg/μL. (**B**): hexanol, (**C**): nonanal, (**D**): (*E*)-2-hexenal, (**E**): (*Z*)-3-hexenol, (**F**): linalool, (**G**): (*E*,*E*)-*α*-farnesene, (**H**): standard reference, (**I**): solvent control. Relative EAG responses are shown as mean ± SE (*n* = 18). Bars indicate standard error. The dashed line represents the stimulation time (0.5 s), the blue vertical line represents the EAG value, and the blue horizontal line represents the time taken for the EAG value to peak.

Among six synthetic compounds of tea volatiles tested, 10 µL of hexane solution of four elicited a noticeable EAG response (relative EAG value ≥ 0.1 mV) at a concentration of 100 µg/µL (Figure 3 and Figure 4), including nonanal (relative EAG value = 0.12 mV) (Figure 3B and Figure 4A), (*Z*)-3-hexenol (relative EAG value = 0.14 mV) (Figure 3D and Figure 4A), linalool (relative EAG value = 0.13 mV) (Figure 3E and Figure 4A), and (*E*,*E*)-*α*-farnesene (relative EAG value = 0.10 mV) (Figure 3F and Figure 4A). The other two compounds, hexanol and (*E*)-2-hexenal, elicited very weak or no response at all tested concentrations (relative EAG value < 0.1 mV) (Figure 3A,C).

### 2.5. Preference of A. spiniferus for Synthetic Compounds of Tea Volatiles

**Figure 5:**
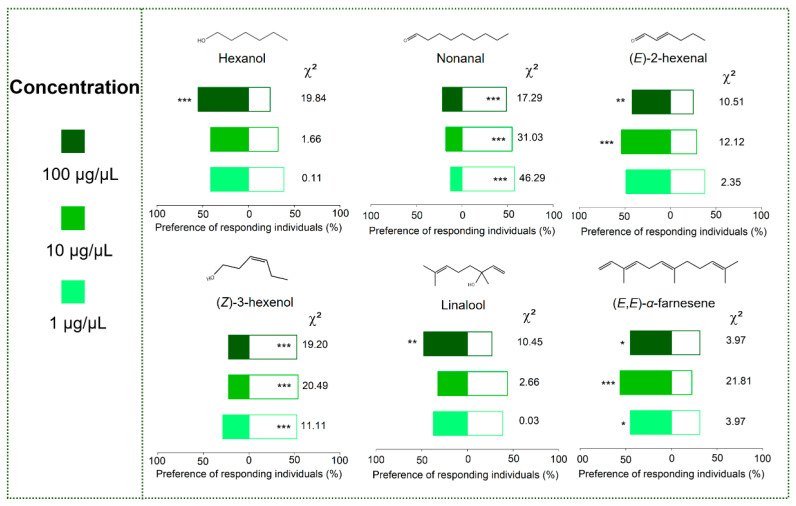
Preference of adult *A. spiniferus* to 10 μL of hexane solution of six tea volatiles at different concentrations (1, 10, and 100 μg/μL). All compounds used in the Y-tube olfactometer assays were diluted with hexane. Asterisks indicate significant (* *p* < 0.05, ** *p* < 0.01, *** *p* < 0.001) preference between the two odor sources by chi-square test.

Compared to the control, 10 µL of hexane solution of six compounds elicited a significant response in adult *A. spiniferus*, with the response being concentration-dependent. Adults were significantly attracted to hexanol at a concentration of 100 µg/µL; linalool at 100 µg/µL; (*E*)-2-hexenal at 100 µg/µL and 10 µg/µL. Similarly, they were attracted to (*E*,*E*)-*α*-farnesene at a concentration of 100 µg/µL, 10 µg/µL, and 1 µg/µL. In contrast, adults were repelled by both nonanal and (*Z*)-3-hexenol at all tested concentrations, which ranged from 1 to 100 µg/µL, compared to hexane (Figure 5).

### 2.6. Field Trials of Synthetic Compounds and Blends of Tea Volatiles

**Figure 6:**
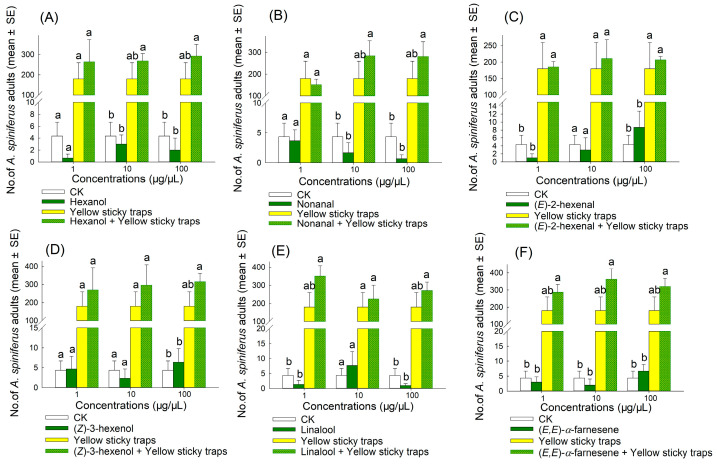
Number of adult *A. spiniferus* in the sticky traps containing 500 μL of hexane solution of six synthetic compounds, hexanol (**A**), nonanal (**B**), (*E*)-2-hexenal (**C**), (*Z*)-3-hexenol (**D**), linalool (**E**), and (*E*,*E*)-*α*-farnesene (**F**), at different concentrations (1, 10, and 100 μg/μL) when the traps were placed after 3 d in Jinan in October 2022. Bars indicate standard error. Different letters above bars indicate significant differences in the number of adult *A. spiniferus* at different treatments, *p* < 0.05, one-way analysis of variance.

**Figure 7:**
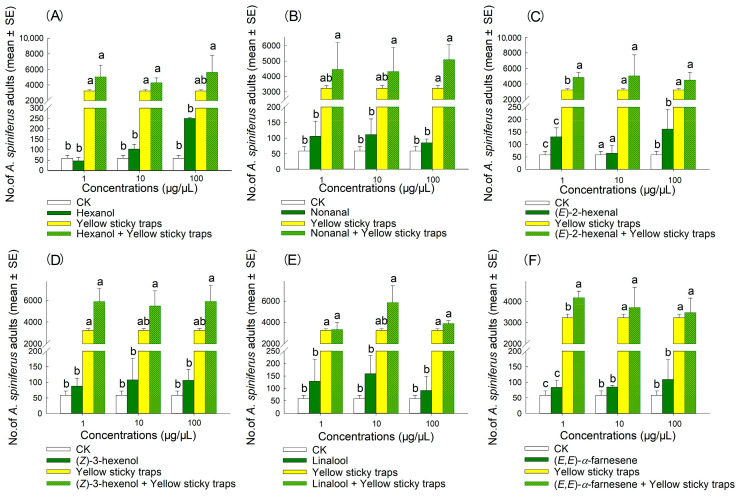
Number of adult *A. spiniferus* in the sticky traps containing 500 μL of hexane solution of six synthetic compounds, hexanol (**A**), nonanal (**B**), (*E*)-2-hexenal (**C**), (*Z*)-3-hexenol (**D**), linalool (**E**), and (*E*,*E*)-*α*-farnesene (**F**), at different concentrations (1, 10, and 100 μg/μL) when the traps were placed after 7 d in Tai’an in October 2023. Bars indicate standard error. Different letters above bars indicate significant differences in the number of adult *A. spiniferus* at different treatments, *p* < 0.05, one-way analysis of variance.

**Figure 8:**
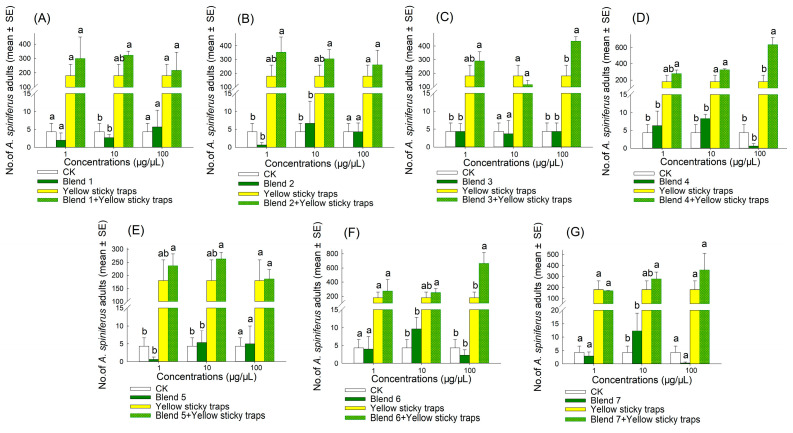
Number of adult *A. spiniferus* caught on sticky traps baited with 500 μL of hexane solution of synthetic blends at different concentrations (1, 10, and 100 μg/μL) when the traps were placed after 3 d in Jinan in October 2022. (**A**) blend 1, (**B**) blend 2, (**C**) blend 3, (**D**) blend 4, (**E**) blend 5, (**F**) blend 6, and (**G**) blend 7. Bars indicate standard error. Different letters above bars indicate significant differences in the number of adult *A. spiniferus* at different treatments, *p* < 0.05, one-way analysis of variance.

**Figure 9:**
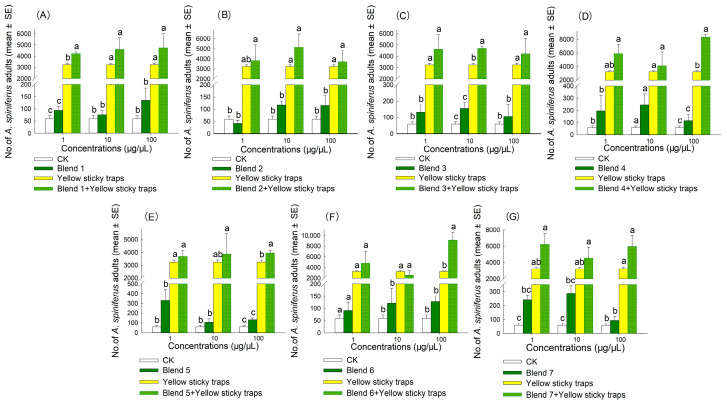
Number of adult *A. spiniferus* caught on sticky traps baited with 500 μL of hexane solution of synthetic blends at different concentrations (1, 10, and 100 μg/μL) when the traps were placed after 7 d in Tai’an in October 2023. (**A**) blend 1, (**B**) blend 2, (**C**) blend 3, (**D**) blend 4, (**E**) blend 5, (**F**) blend 6, and (**G**) blend 7. Bars indicate standard error. Different letters above bars indicate significant differences in the number of adult *A. spiniferus* at different treatments, *p* < 0.05, one-way analysis of variance.

The result indicated that the synergistic bait trap (comprising an attractant and a yellow sticky trap) produced the best attraction effect (Figure 6, Figure 7, Figure 8 and Figure 9). In 2022, the trapping effectiveness of synergistic bait traps containing blend 3 at 100 µg/µL (Figure 8C), blend 4 at 100 µg/µL (Figure 8D), and blend 6 at 100 µg/µL (Figure 8F) was significantly higher than that of the other three treatments (CK, single attractant, and yellow sticky trap). In 2023, synergistic bait traps containing (*E*)-2-hexenal at 1 µg/µL (Figure 7C), (*E*,*E*)-*α*-farnesene at 1 µg/µL (Figure 7F), blend 1 at 1 µg/µL (Figure 9A), blend 3 at 10 µg/µL (Figure 9C), blend 4 at 100 µg/µL (Figure 9D), blend 5 at 100 µg/µL (Figure 9E), and blend 6 at 100 µg/µL (Figure 9F) were all significantly more effective than the other three treatments.

**Figure 10:**
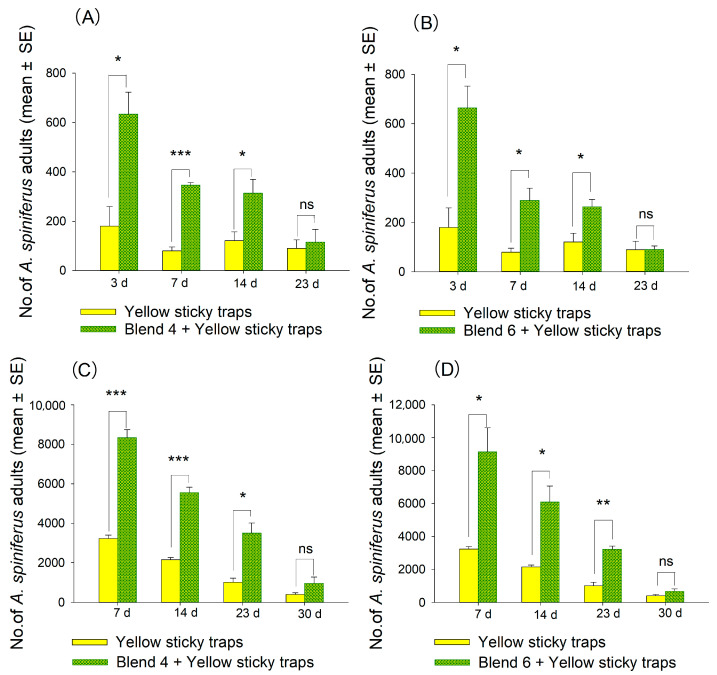
Number of adult *A. spiniferus* in yellow sticky traps containing 500 μL of hexane solution of blend 4 at 100 μg/μL and blend 6 at 100 μg/μL at different times after application in 2022 (**A**,**B**) and 2023 (**C**,**D**). Bars indicate standard error. Different asterisks above bars indicate significant differences in the number of adult *A. spiniferus* in the different treatments. * *p* < 0.05; ** *p* < 0.01; *** *p* < 0.001; ns indicates non-significant difference. All experiments were performed in three replicates, two-tailed Student’s *t*-test.

Due to the excellent performance of blend 4 [the mixture of linalool and (*E*,*E*)-*α*-farnesene] and blend 6 [the mixture of (*Z*)-3-hexenol and linalool] at a concentration of 100 µg/µL over two consecutive years, we analyzed their lure longevity time. From 3 to 14 days after application in 2022 and from 7 to 23 days after application in 2023, the number of adult *A. spiniferus* captured in yellow sticky traps containing these two blends was significantly higher than those using yellow sticky traps alone (Figure 10). Between 14 to 23 days after application in 2022 and from 23 to 30 days after application in 2023, there was no significant difference in the number of adults captured by the two treatments compared to the yellow sticky trap alone (Figure 10).

## 3. Discussion

In this study, adult *A. spiniferus* showed a preference for ‘Huangjinya’ and ‘Fuding white tea’ over ‘Baiye No. 1’ and ‘Longjing 43’, and laid more eggs in the same environment of a tea plantation (Figure 1). ‘Huangjinya’ is notably susceptible to common pests of tea plants [43,45], while ‘Fuding white tea’ offers higher free amino acid content, marking it more suitable for herbivorous insects [44]. Although the nutritional level of plants often affects their suitability and resistance to herbivorous insects, insects initially locate distant host plants using their sense of smell and vision [21,48].

External morphological features of tea plants may be an important factor in the attraction of adult *A. spiniferus*, such as the unique yellowish-green leaves of ‘Huangjinya’, which appeal more to insects with a preference for yellow color [49]. However, indoor Y-shaped olfactometer experiments indicated that adult *A. spiniferus* remained strongly attracted to both ‘Huangjinya’ and ‘Fuding white tea’ even without visual cues, highlighting the crucial role of olfaction in their host-finding process.

When presented with healthy plants and whitefly-infested plants of ‘Longjing 43’ (which this study identified as a resistant variety), 62.5% of adults did not express a preference (Figure 2A). This suggests that adult *A. spiniferus* may dislike the odor of ‘Longjing 43’, leading to its avoidance in the field. Herbivores often display a distinct preference for certain plant varieties based on odor. For instance, zingiberence and curcumene, two sesquiterpene volatile compounds found in wild tomato varieties resistant to B. tabaci, but absent in susceptible cultivated varieties, are highly repellent to B. tabaci and have an effect on the host selection of this pest [50]. This underscores the importance of plant odor, which is critical to resistance or susceptibility. It is, therefore, crucial to identify the key active components that mediate host selection.

GC-MS was used to analyse and identify the composition of tea volatiles. In this study, (*E*,*E*)-*α*-farnesene was detected at 34.25 ± 2.6 (ng plant^−1^ h^−1^) in ‘Huangjinya’ (Table 2). This compound exhibited significant attractive effects at the concentrations of 1, 10, and 100 µg/µL (Figure 4). (*E*,*E*)-*α*-farnesene is a common herbivore-induced volatile present in many plants, and serves as a positive signal for attracting natural enemies [29,51]. Thus, its presence may have both positive and negative effects, as it attracts natural enemies while making the tea plant vulnerable to attack by *A. spiniferus*.

Except for the susceptible ‘Huangjinya’, (*Z*)-3-hexenol was found in the headspace volatiles of the other three cultivars (Table 2). Laboratory tests confirmed that (*Z*)-3-hexenol had a repellent effect (Figure 5). It is released by tea plants as a defense mechanism against herbivores [38,52,53]. This compound contributes to the distinctive grassy aroma of green tea [54].

Nonanal, another verified repellent (Figure 5), was not detected in the resistant variety ‘Longjing 43’ (Table 2). Conversely, attractive compounds like hexanol and (*E*)-2-hexenal were more abundant in the headspace volatiles of resistant ‘Longjing 43’ and ‘Baiye No. 1’ (Figure 5 and Table 2). A possible explanation is that most insects typically utilize mixed volatiles for host identification rather than single components [21]. For example, female *Aulacphora foveicollis* (Coleoptera: Chrysomelidae) reacted positively to the volatile mixture of creeping cucumber fruits due to the synergistic effect of nonanal and (*E*,*Z*)-2,6-nonadienals [55]. Therefore, when applying these compounds in the field, it is essential to consider the potential synergistic effect of combining them. It should be noted that due to the different gain of mass spectrometers for each compound, using different models of mass spectrometers may give different “mean emission amounts”.

The antennae of insects are one of the most important olfactory organs; EAG experiments can clarify their role in sensing external chemical stimuli [56,57]. Although hexanol and (*E*)-2-hexenal did not elicit a strong EAG response (Figure 3A,C), adult *A. spiniferus* displayed positive behavioral responses to these compounds (Figure 5). It is widely recognized that various tissues of insects are involved in chemical sensing, as such chemoreceptors are present in body hairs, appendages, mouthparts, and wings of insects [58]. Odorant-binding proteins (OBPs) and chemosensory proteins (CSPs), which play a role in binding and transporting odor molecules, are also expressed in tissues outside the antennae [59,60]. Tissue-specific transcriptome analysis indicated that CSPs are widely expressed in both the head (including the antennae) and body tissues of adult *A. spiniferus* [61]. Therefore, it is hypothesized that the behavioral responses to specific chemical signals may occur without a clear EAG response.

It would be a major breakthrough if active compounds from tea tree volatiles could be used to control this pest in the field. The field trial found that two combinations of attractants based on tea volatiles, blend 4 [the mixture of linalool and (*E*,*E*)-*α*-farnesene (3:1, *v*/*v*)] and blend 6 [the mixture of linalool and (*Z*)-3-hexenol (3:1, *v*/*v*)], were found to be highly effective and long lasting in trapping adult *A. spiniferus* over two years (Figure 8D,F, Figure 9D,F and Figure 10). Moreover, these blends outperformed single components. This finding aligns with previous studies on other pests [35,37]. Field trials also revealed a significant improvement in trapping efficiency when combined with yellow sticky traps, suggesting that the addition of baits may enhance the effectiveness of traps based on visual cues. The strategy of combining visual and olfactory stimuli has been implemented in managing the citrus psyllid [62]. It is noteworthy that more attractants demonstrated a substantial luring effect in the tea plantation with a high density of adult *A. spiniferus*. Previous studies indicated that when populations were low, expanding the entry area can increase captures and improve selectivity for *Drosophila suzukii* [63]. In areas severely affected by dense bark beetle populations, the use of anti-attractants proved ineffective [64]. This underscores the importance of considering population density when assessing the effectiveness of an attractant.

In the lure longevity experiment conducted in October 2023, the number of adult *A. spiniferus* trapped by yellow sticky traps gradually declined (Figure 10), consistent with results from population dynamics (Figure 2A). The field trial took place during the third peak of *A. spiniferus* adult occurrence, after which the number of adults steadily decreased. This indicates that the longevity of the lure may be influenced by seasonal factors, necessitating long-term monitoring tests [65]. (*Z*)-3-hexenol, linalool, and (*E*,*E*)-*α*-farnesene, the core compounds of mixed attractant blends 4 and 6, have been proven to exhibit certain sustained release effects when adsorbed on an agricultural rubber dispenser. (*Z*)-3-hexenol remains effective for at least 3 weeks in specific sustained-release lures [66], linalool can achieve sustained release for several weeks through microcapsules and other means [67], and *α*-farnesene may have relatively long-lasting release due to its structural properties [68], though relevant studies are scarce. A 2021 study revealed that when tea plants are fed upon by *E. obliqua* larvae, they release volatiles including (*Z*)-3-hexenol, linalool, and (*E*,*E*)-*α*-farnesene. These compounds trigger neighboring tea plants to emit *β*-ocimene, thereby enhancing repellent effects against adult *E. obliqua* moths [69]. Functionally, linalool attracts pollinators [28], while (*Z*)-3-hexenol is rapidly released in large quantities when plants suffer herbivorous insect damage, serving as an immediate stress response signal [70]. This functional divergence may result in counteracting effects between the two compounds. In summary, complex synergistic or antagonistic interactions exist among these three compounds, leading to distinct attraction effects in their blended formulations. In addition, *A. spiniferus* is distributed in an even way during a large-scale outbreak [71]. The test tea plantation experienced a large-scale outbreak of *A. spiniferus* in 2023, with 8000 to 10,000 individuals captured by blends 4 and 6 within 7 days (Figure 10), avoiding the impact of spatial bias. Therefore, the field trial in 2023 appears to have a higher practical reference value.

The field trials revealed two anomalies: (i) (*Z*)-3-hexenol and nonanal, which acted as repellent in the laboratory test, showed positive response in field experiments; (ii) in Tai’an (2023) but not Jinan (2022), the lowest dose of (*E*)-2-hexenal, (*E*,*E*)-*α*-farnesene, and blend 1 increased catches significantly, contradicting laboratory findings. This inconsistency may result from multiple factors, including volatile degradation [72], microclimate [73,74], wind dispersion [75,76], and background odors [77]. Field conditions, unlike the stable lab environment, accelerate volatile breakdown due to sunlight, oxygen, and microorganisms, altering their composition [78]. Microclimate factors like temperature and humidity affect volatile emission, as seen in studies on apple twigs [74]. Wind disperses odors, changing concentration and gradients, with short-distance ratio variations [74]. Background odors in the field interfere with the attractiveness of plant volatiles to herbivorous insects [77]. Similar observation was made in a study screening *D. suzukii* attractant volatiles, where the co-attractiveness of certain compounds varied under different background odor environments, suggesting that background odor can influence detection of potential attractants [79]. In another study, five traps were placed at different sites in a corn field where *Anomala corpulenta* (Coleoptera: Scarabaeoidea) had emerged, but only the baits at sites 1–4 effectively lured and trapped them [80]. This suggests that for more accurate conclusions, field trials should be conducted at various locations while considering the impact of background clues. Although this study was validated in the tea plantation of Shandong Province, future experiments will be conducted in different geographical regions to verify the effectiveness of VOC mixtures under different climatic and ecological conditions. The current research is limited to the attractant effect of *A. spiniferus*; further attention should be paid to the number and identity of non-target species in traps.

## 4. Materials and Methods

### 4.1. Tea Cultivars, Insect Collection, and Chemicals

Four tea cultivars, ‘Baiye No. 1’, ‘Longjing 43’, ‘Fuding white tea’, and ‘Huangjinya’, were used for headspace volatile analysis, behavioral preferences assessment, and population dynamics of adult *A. spiniferus*. They were cultivated in the same tea plantation managed by Shandong Qianrun Ecological Agriculture Development Co., Ltd. in Tai’an, Shandong Province, China (32°08′ N, 117°43′ E). The tea plantation covers an area of approximately 50 ha, with four tea plant varieties cultivated in roughly equal proportions, spaced at intervals exceeding 10 m. All tea cultivars were grown under identical conditions in the same field and received uniform management practices, including standardized fertilizer application (type, rate, timing, method), irrigation, pest control, and other agronomic measures.

Adult *A. spiniferus* were collected from the ‘Huangjinya’ cultivar maintained in the tea plantations, as described above. Laboratory tests were conducted in environmental incubators at 26 ± 2 °C with 70 ± 5% relative humidity (RH) and a 16L:8D photoperiod. When two cultivars were tested for preference, the test insects remained on the corresponding cultivars for 48 h. Insects for the other laboratory tests were kept on ‘Huangjinya’. Due to the low male-to-female ratio, we combined both sexes for subsequent experiments.

Hexane (analytical pure) served as the solvent, and the negative control was purchased from Tianjin Kaitong Chemical Reagent Co. Ltd. (Tianjin, China). Hexanol (analytical pure, 99%, CAS: 111-27-3), linalool (analytical pure, 98%, CAS: 78-70-6), (*Z*)-3-hexenol (analytical pure, 98%, CAS: 928-96-1), nonanal (analytical pure, 96%, CAS: 124-19-6), (*E*)-2-hexenal (analytical pure, 98%, CAS: 6728-26-3), and (*E*,*E*)-*α*-farnesene (analytical pure, 96%, CAS: 21499-64-9) were purchased from Macklin Inc. (Shanghai, China).

### 4.2. Population Dynamics of A. spiniferus on Four Tea Cultivars

The experiment was conducted in the same tea plantations as described above from May to November 2022. Adult *A. spiniferus* typically aggregate towards tender leaves and buds, being mainly active on the upper branches of the tea plants; nymphs and pseudopupae are mainly active on the middle and lower leaves [71]. Therefore, the second and third leaves below the bud were selected for counting the density of adults and eggs in the four tea cultivars. We also investigated the number of nymphs and pseudopupae on the middle and lower leaves. Specifically, a sampling point (5 m × 5 m) was randomly selected as one replicate; one hundred leaves in each sampling point were randomly selected for counting the number of *A. spiniferus* adults, eggs, nymphs, and pseudopupae as a biological replicate, respectively. There were four replicates for each tea cultivar.

### 4.3. Y-Tube Test for Behavioral Responses of Adult A. spiniferus to Tea Shoots and Synthetic Volatile Compounds

The Y-tube olfactometer test was refined based on related studies [81]. Bioassays were conducted using a dual-choice glass Y-tube olfactometer, which consisted of a central tube and two lateral arms (15 cm long, 13 mm diameter) with an inside angle of 60°. For each paired bioassay, two different odor sources were placed in two odor bottles, which were connected to an activated carbon-filtered, humidified air source and the two arms of a Y-tube through Teflon^®^ tubing. The airflow rate through each arm was 100 mL/min by the pre-experiment, measured using an LZB-3WB rotor meter (Yuyao Zhenxing Instrument Factory, Yuyao, China).

Two-year-old tea plants were used for tea plant testing (the growth conditions are described in Section 4.1). Two treatments were set up in the experiment: healthy plants and whitefly-infested plants. Pretreatment for whitefly-infested plants was as follows: eight healthy potted tea plants were placed in a nylon screen cage (50 cm × 50 cm × 50 cm) with 500 (±10) *A. spiniferus* adults per plant for each cultivar. Adults were removed when 200 (±10) eggs were observed on each plant. Eggs in tea plants were reared in environmental incubators at 26 ± 2 °C with 70 ± 5% RH and a 16L:8D photoperiod. Tea plants were used when eggs had developed to the 2nd-3rd instar. Eight healthy potted tea plants for each cultivar were placed in a nylon screen cage (50 cm × 50 cm × 50 cm) as a healthy plant treatment.

To prepare the test chemicals, six standard synthetic chemicals were dissolved in hexane at three different concentrations (1, 10, and 100 µg/µL) in the pre-experiment. Odorants (10 µL) were added dropwise to filter paper strips (Newstar Co. Ltd., Hangzhou, China, 2 × 2 cm), then allowed to evaporate for 20 s and placed in an odor bottle. Filter paper strips (2 × 2 cm) containing hexane were placed in another odor bottle.

Experimental adults were starved for 1 h before the bioassay. A total of 80 insects were tested per bioassay, and each individual was only used once. In each test, a choice was recorded if the adult moved 5 cm past the Y-junction within ten minutes and remained there for at least one minute. Otherwise, it was recorded as no-choice. To avoid the position effect, the positions of the two odor bottles are swapped for every 10 groups tested. At the end of the odor source test, the Y-tube, odor bottles, and other glass components were cleaned with acetone and dried before reuse. The Y-tube olfactometer was placed in the laboratory with diffuse light (3000 lux) at a temperature of 26 ± 2 °C and a relative humidity of 70% ± 5% at about 09:30–15:30 hrs.

### 4.4. Volatile Collection and Analysis

Headspace volatiles from tea plants were collected using the dynamic headspace absorption technique [82]. Healthy tea plants were used in subsequent experiments, as the presence or absence of *A. spiniferus* did not affect the preference of subsequent adults. Six 2-year-old healthy potted tea plants without nymphs were placed in the sealed glass cylinder chamber (50 cm high, 20 cm in diameter). They were planted in the controlled environment chamber at 26 ± 2 °C with 70 ± 5% RH and a 16L:8D photoperiod prior to testing. An empty pot without tea plants was used as the control, and the final results had removed compounds from the control. Each tea cultivar was repeated four times, with new tea plants replaced in each replication. All tea plant samples were strictly harvested at the standardized phenological stage of ‘two leaves and a bud’ in early spring. The inlet, outlet, and all interfaces of the glass cylinder chamber were sealed with sealing film (Bemis, Inc., Neenah, WI, USA). The airflow entered the glass cylinder chamber after passing through the silicone desiccant, activated carbon, and rotameter at 200 mL/min. Volatiles were extracted after collection onto the glass tube containing 35 mg of Super Q absorbent (35 mg; 80/100 mesh; Alltech Associates., Inc., Deerfield, IL, USA). All components of the volatile collection apparatus were connected by Teflon^®^ tubing. After 1 h of collection, the trapped tea shoot volatiles were extracted from the Super Q tube with chromatographically pure hexane (500 µL). The eluting solution was stored in a glass vial to which ethyl decanoate (5 µL) at a concentration of 10^−4^ g/mL was added as an internal standard. Ethyl decanoate was commonly used as internal standard in the identification of tea volatiles [52,53,83]. The sample was stored at −20 °C for qualitative and quantitative analysis of volatile components.

The gas chromatography (GC) analysis was performed using an Agilent 7890B model, and the mass spectrometer (MS) used was the Agilent 7000D model (Agilent, Inc., PaloAlto, CA, USA). Samples were analyzed on an HP-5ms (30 m × 0.25 mm i.d., 0.25 µm film thickness). The solvent delay was set to 3 min, the inlet temperature was 230 °C, and the GC/MS interface temperature was 280 °C. The analysis program consisted of an initial oven temperature programmed at 40 °C for 0 min, followed by an increase at 3 °C/min to 190 °C and held for 2 min, and then increased at 10 °C/min to 290 °C, held for 3 min. Helium (99.999% purity) was used as the carrier gas at a flow rate of 1 mL/minute. The mass spectrometer was operated in the electron-impact (EI, at 70 eV) scan mode. The mass scan range was 35 to 350 *m*/*z*. The scan rate was twice per second. Qualitative analysis of the volatile components was processed by Agilent MassHunter Qualitative Analysis Navigator software (B.08.00).

Analysis and quantification of compounds refer to previous research [24,84]. Compounds were identified by comparing the obtained spectra with the spectra of reference compounds from NIST17. Retention indices (RIs) of volatile compounds from tea plants were then calculated according to the retention time of n-alkanes (C4-C30). Peaks were assigned when the MS similarity score was ≥80% and the RI difference was <10. The contents of each volatile compound were calculated by comparing their GC total ion current peak areas with the peak area of the internal standard (ethyl decanoate).

The formula for calculating the retention index is:

$$\mathrm{RI}\;=\;100n+\;100\;\times \;\frac{T_{t}\;-\;T_{tn}}{T_{t(n+1)}\;-\;T_{tn}}$$

*n* and *n*+1 represent the carbon atom numbers of the n-alkanes eluted consecutively in the chromatographic analysis; *T_tn_* and *T_t_*_(*n*+1)_ are the retention times of the corresponding n-alkanes; *T_t_* is the retention time of volatile components in the sample, satisfying (*T_tn_* < *T_t_* < *T_t_*_(*n*+1)_)/min.

### 4.5. Electroantennogram Experiments

The EAG test was conducted using the same method as the determination of antennally active volatiles for tea geometrids [25]. The EAG system (Syntech, Ltd., Hilversum, The Netherlands) consisted of an IDAC-4 interface box, a CS-55 air stimulus controller, and a desktop computer.

Hexane is commonly used to dissolve small compounds in research [85,86]. Six standard chemical compounds were dissolved in hexane at five different concentrations (1, 10, 100, 250, and 500 µg/µL). Exactly 10 µL of the tested solution was placed onto a filter paper strip (0.5 × 6 cm), the solvent was allowed to evaporate for 30 s, then the strip was carefully transferred to a Pasteur pipette cartridge (14.5 cm long). Hexane (10 µL) was used as the control using the same method.

The adult *A. spiniferus* was chilled in an ice box and removed immediately after the induction of torpor. An antenna was dissected under the stereoscopic microscope (Nikon, Ltd., Japan), and then the reference and recording electrodes were attached to the base and tip of the antenna using conductive adhesive, respectively. To perform the stimulation, the tip of a Pasteur pipette cartridge was inserted into a small hole in the wall of a steel tube (14 cm long, 8 mm diameter), approximately 3 mm in diameter, located approximately 11 cm from the end of the tube. A regulated airflow (10 mL/s) entered the steel tube and was directed toward the antenna for chemical exposure. The antenna was located approximately 1 cm from the end of the steel tube. Each antenna was randomly stimulated for testing with three tested chemicals. The stimulation sequence consisted of a solvent control (hexane) stimulus, standard reference (3-carene), followed by three test chemical stimuli, and then standard reference, solvent control. Low-concentration compounds were prioritized for testing. The duration of each stimulation was set to 0.5 s, with an interval of 60 s between two consecutive stimuli [87]. The relative EAG value is the difference between the chemical and the solvent control. A total of 18 replicates were measured for each concentration level of volatiles. Only one antenna was randomly selected per adult. EAG tests were conducted at a temperature of 26 ± 2 °C and a relative humidity of 70 ± 5% at 09:30–15:30 h.

### 4.6. Field Trial

The field trial was conducted in October 2022 in the tea plantation in Gongjiazhuang Village, Jinan City, Shandong Province, China (36°31′ N, 117°41′ E), where there was a low density of *A. spiniferus* adults. In October 2023, the trial took place at the same tea plantation as described in the population dynamics experiment, where there was a high density of *A. spiniferus* adults. The implementation time coincided with the third peak of *A. spiniferus* adult occurrence in tea plantations. The average temperature and relative humidity during the field trials were 8–15 °C and 55–70% RH, respectively, with sunny or cloudy weather and a gentle breeze (data source: http://data.cma.cn/, accessed on 1 December 2023). Considering the volatile components in susceptible cultivars, along with physicochemical and physiological characteristics of compounds, the following seven blends were selected (Table 2).

The attractants for tests were formulated as six single-component and seven different mixed-component blends diluted with hexane to three concentrations (1, 10, and 100 µg/µL) and adsorbed onto a rubber dispenser (Keyun, Co. Ltd., Henan, China), each containing 500 µL. The following treatments were included: (i) synergistic bait traps: attractants + yellow sticky traps (25 × 20 cm, Lvpusen Technology, Ltd., Quanzhou, China); (ii) yellow sticky traps; (iii) single attractants: attractants + white sticky traps (25 × 20 cm, Lvpusen Technology, Ltd., Quanzhou, China), with the white sticky trap used as a control.

All sticky traps were attached to bamboo poles and placed 5–10 cm from the tea shoots; the spacing was 25 m between each trap to minimize the interference between compounds. The trial was conducted in three plots of approximately 1 ha each, with the four treatments randomized between plots. The distance between the two plots was at least 15 m. The number of adult *A. spiniferus* on sticky traps was counted on days 3, 7, 14, and 23 in 2022 and on days 7, 14, 23, and 30 in 2023 following the start of the experiment. The sticky traps were replaced after each survey. Due to the large-scale outbreak of *A. spiniferus* in the experimental tea plantation in 2023, we extended the statistical time accordingly.

### 4.7. Data Analysis

All data were analyzed in GraphPad Prism 9.0.0.121 software (GraphPad Software, San Diego, CA, USA). Data on population dynamics, volatile quantities, relative EAG response, and field trials were analyzed by one-way analysis of variance (ANOVA). The normality of the residuals was tested for all data using the Shapiro–Wilk test. Means were compared using Tukey’s-b multiple range test or the Games–Howell test, depending on whether the treatment and control variances were equal (Shapiro–Wilk test, *p* > 0.05) or unequal (Shapiro–Wilk test, *p* < 0.05). The Y-tube olfactometer behavioral test data were analyzed using a chi-squared test (*p* < 0.05) to determine differences between pairs of treatments. Non-choice individuals were recorded but excluded from the statistical analysis. Data on lure longevity time was analyzed by a two-tailed Student’s *t*-test.

## 5. Conclusions

In conclusion, our study demonstrates that ‘Huangjinya’ and ‘Fuding white tea’ were more attractive and heavily infested by *A. spiniferus*; ‘Baiye No. 1’ and ‘Longjing 43’ are resistant cultivars. Specific compounds of tea volatiles are involved in the host selection of adult *A. spiniferus* among tea cultivars. The two effective and long lasting combination of attractants based on tea volatiles, 500 µL of hexane solution of the mixture of linalool and (*E*,*E*)-*α*-farnesene (3:1, *v*/*v*), and the mixture of linalool and (*Z*)-3-hexenol (3:1, *v*/*v*) at the concentration of 100 µg/µL, are promising candidates for developing plant-derived lures for IPM applications.

## Figures and Tables

**Table 1:** Identified volatile organic compounds (VOCs) emitted from four tea cultivars (‘Huangjinya’, ‘Fuding white tea’, ‘Baiye No. 1’, and ‘Longjing 43’) and mean emission amount * (ng plant^−1^ h^−1^).

Volatile Compounds	CAS	Retention Time (min)	Retention Index ^†^	Retention Index ^‡^	Concentration (ng plant^−1^ h^−1^)			
					Baiye No. 1	Fuding White Tea	Huangjinya	Longjing 43
Hexanol	111-27-3	3.21	859	863	6712 ± 562 a	3728 ± 959.3 ab	1668 ± 322.8 b	5634 ± 688.4 a
(*E*)-2-Hexenal	6728-26-3	7.21	867	868	235.8 ± 49.13 ab	167.6 ± 21.43 ab	90.83 ± 21.33 b	249.5 ± 37.39 a
(*Z*)-3-Hexenol	928-96-1	7.42	875	877	48.28 ± 4.37 a	29.11 ± 4.10 a	ND	32.96 ± 4.83 a
Benzaldehyde	100-52-7	8.43	957	956	567.6 ± 124.8 a	406 ± 84.73 a	172.9 ± 88.29 a	235.6 ± 35.35 a
2-Ethyl-1-hexanol	104-76-7	11.80	1031	1038	228.9 ± 64.03 a	251 ± 51.94 a	220.9 ± 61.29 a	425.1 ± 67.08 a
(*E*)-*β*-Ocimene	3779-61-1	12.94	1052	1058	1045 ± 398.3 a	789.5 ± 158.5 a	566 ± 154.6 a	540 ± 169.5 a
Linalool	78-70-6	15.62	1082	1075	ND	96.29 ± 23.63	ND	ND
Nonanal	124-19-6	15.93	1102	1095	60.37 ± 18.47 a	34.35 ± 4.22 a	14.54 ± 3.72 a	ND
(*Z*)-3-Hexenyl butanoate	16491-36-4	19.11	1179	1186	1025 ± 248.8 a	858.8 ± 149.7 a	689 ± 179.8 a	784.7 ± 237.2 a
7-Methyl-heptadecane	20959-33-5	26.62	1334	1330	681.6 ± 202 b	636 ± 82.64 b	1816 ± 137.9 a	1356 ± 336.4 ab
(*E*,*E*)-*α*-Farnesene	21499-64-9	30.69	1505 ^§^	1510	ND	ND	34.25 ± 2.60	ND
Cadalene	483-78-3	40.56	1673	1674	814.2 ± 256.8 a	963.2 ± 174.4 a	766.7 ± 160 a	1714 ± 303.5 a
Caffeine	58-08-2	46.29	1840	1835	227.8 ± 47.99 a	303.4 ± 41.73 a	252.3 ± 50.87 a	415.1 ± 64.31 a

* Data in the table are presented as mean ± SE (*n* = 3), and those followed by different letters in the same row indicate significant differences at 0.05 level by one-way analysis of variance, Tukey’s-b multiple range test. “ND” denotes that compounds were not detected. These entries were excluded from all statistical calculations. ^†^ Retention index were found in the NIST database (https://webbook.nist.gov/chemistry/cas-ser/ (accessed on 17 July 2025)), and were obtained on a DB-5 or HP-5 column. **^‡^** Retention index determined on HP-5MS column using the homologous series of n-alkanes (C4−C30). ^§^ The retention index was obtained from Khan et al. [47].

**Table 2:** Composition of seven blends formulated as potential attractants for adult *A. spiniferus*.

Code	Component	Ratio
Blend 1	Hexanol, nonanal, (*E*)-2-hexenal, (*Z*)-3-hexenol, linalool, (*E*,*E*)-*α*-farnesene	55:1:3:1:3:1 (*v*/*v*/*v*/*v*/*v*/*v*)
Blend 2	Hexanol, (*Z*)-3-hexenol, linalool, (*E*,*E*)-*α*-farnesene	55:1:3:1 (*v*/*v*/*v*)
Blend 3	Nonanal, (*E*)-2-hexenal	1:3 (*v*/*v*)
Blend 4	(*E*,*E*)-*α*-farnesene, linalool	1:3 (*v*/*v*)
Blend 5	(*Z*)-3-hexenol, (*E*,*E*)-*α*-farnesene	1:1 (*v*/*v*)
Blend 6	(*Z*)-3-hexenol, linalool	1:3 (*v*/*v*)
Blend 7	Hexanol, (*Z*)-3-hexenol	55:1(*v*/*v*)

## Data Availability

Data will be made available on request.
